# A global drought dataset for Multivariate Composite Drought Index (MCDI) and its constituent drought indices

**DOI:** 10.1038/s41597-025-06320-x

**Published:** 2025-11-24

**Authors:** Mengjia Yuan, Guojing Gan, Jingyi Bu, Yanxin Su, Hongyu Ma, Xianghe Liu, Leyao Zhang, Yongqiang Zhang, Yanchun Gao

**Affiliations:** 1 Key Laboratory of Water Cycle and Related Land Surface Processes, Institute of Geographic Sciences and Natural Resources Research, Chinese Academy of Sciences, Beijing, 100101 China; 2 University of Chinese Academy of Sciences, Beijing, 100049 China; 3 State Key Laboratory of Lake and Watershed Science for Water Security, Nanjing Institute of Geography and Limnology, Chinese Academy of Sciences, Nanjing, 211135 China; 4 Earth Systems Research Center, Institute for the Study of Earth, Oceans, and Space, University of New Hampshire, Durham, NH 03824 USA

**Keywords:** Hydrology, Natural hazards, Hydrology, Climate change

## Abstract

High-resolution drought index datasets are essential for drought monitoring and assessment. Despite numerous global/regional drought index datasets, the composite drought index datasets that comprehensively consider meteorological, agricultural, and hydrological factors are still very few, thus hindering the capture of complex drought dynamics and comprehensive risk assessment. To address this gap, this study produced a 0.1° resolution global drought dataset (1980–2019) based on the newly developed concept of Multivariate Composite Drought Index (MCDI), which considered the time lag and cumulative effects of drought and could characterize comprehensive drought characteristics effectively. The dataset contains MCDI and its four constituent indices (Standardized Precipitation Actual Evapotranspiration Index (SPAEI), Standardized Soil Moisture Index (SSI), Standardized Runoff Index (SRI), Water Storage Deficit Index (WSDI)) on a monthly scale. Verification results showed that they indicated the drought evolution and ecosystem response process well, especially the MCDI. Overall, the dataset compensated for the data deficit of the comprehensive drought index and would provide data support for global drought monitoring and adaptive management of drought under climate change.

## Background & Summary

Drought is a natural hazard triggered by intense and persistent water deficits with relatively wide-ranging impacts^1–3^. Severe drought events can impede crop and vegetation growth, threaten ecosystem health, disrupt normal livelihoods, and cause significant socio-economic losses^4–6^. Moreover, these impacts are often unevenly distributed, disproportionately affecting vulnerable populations and thereby exacerbating global health, well-being, and gender inequalities^7^. Under the combined pressures of climate change and human activities, drought will become more frequent, severe, and unpredictable in many regions of the world^8–10^. Given its extensive reach and destructive potential, drought has garnered widespread attention from various sectors in recent years, which has also highlighted the urgency of developing quantitative drought indicators and establishing corresponding datasets for drought monitoring^5,11,12^.

Drought index is one of the most widely used and effective tools to quantitatively characterize drought^13^. The existing drought indices can be broadly categorized into drought indices constructed for specific types of droughts (e.g., meteorological drought, agricultural drought, etc.) and composite drought indices for a comprehensive assessment of drought^14,15^. The first type of drought indices tends to rely on environmental variables related to a specific type of drought. For example, the Standardized Precipitation Index (SPI)^16^, Standardized Soil Moisture Index (SSI)^17^, and Standardized Runoff Index (SRI)^18^ are often used to characterize meteorological, agricultural, and hydrological drought, respectively. This is due to the fact that they are constructed solely based on precipitation (P), soil moisture (SM), and runoff (RO), respectively. Composite drought indices, on the other hand, are indices that combine more environmental variables or different types of univariate drought indices^19–21^. For example, Zhang, *et al*.^5^ constructed a multivariate standardized drought index (MMSDI) by integrating P, evapotranspiration (ET), and SM, finding that MMSDI had advantages in agricultural drought detection in China. Based on the Gaussian copula function, Shah and Mishra^6^ developed an Integrated Drought Index (IDI) by integrating the SPI, SRI, SSI, and standardized groundwater index (SGI), finding that IDI could effectively monitor and assess drought under retrospective and future climates in India. Compared to the first type of drought indices, the composite drought indices can better reflect the characteristics of integrated, complex drought events^15,22,23^.

As droughts become more frequent, many studies have pointed to the need to consider the lagging and cumulative effects of drought in drought research-related work^6,24–26^. This is because the frequency and severity of droughts may lead to the normalization of one drought event before the end of another, leading to the gradual collapse of ecosystems^27–29^. Accounting for the effects of previous droughts and the environmental context in which droughts occur can better reflect the ecosystem response to drought and provide a scientific basis for drought monitoring and early warning^25^. To make up for the fact that the existing composite drought indices seldom consider the lagged and cumulative effects of drought, Yuan, *et al*.^30^ recently proposed a new composite drought index named the Multivariate Composite Drought Index (MCDI). Based on the Gringorten empirical formula, MCDI integrated the meteorological drought index (Standardized Precipitation Actual Evapotranspiration Index (SPAEI)), agricultural drought index (SSI), and hydrological drought indices (SRI, Water Storage Deficit Index (WSDI)) with accounting for the time lag between different types of droughts and the cumulative effects of droughts. The application results in China showed that MCDI has good potential for drought monitoring and assessment, and this is also the drought index used in this study to construct the global drought dataset.

The exploration of drought indices emphasizes the need for high-quality drought datasets, and the lack of consistent source data increases the difficulty of quantifying drought^3,31^. Many global/regional drought datasets have been generated, such as self-calibrating Palmer Drought Severity Index (scPDSI)^32–34^, SPEI^13,35–37^, and SPI^3,16,38^ datasets with different spatial-temporal resolution. It can be noted that although many drought index datasets have emerged globally and regionally, there are still very few composite drought index datasets. This means that many of the ideas for constructing a composite drought index have not been converted into realistic datasets, which would be unfavorable for methodological development and research. Firstly, the lack of a composite drought index dataset is equivalent to the loss of a means of comprehensively assessing drought. Secondly, the lack of a composite drought index dataset would be detrimental to the emergence of new composite drought indices and their assessment. Specifically, when a new composite drought index appears, it cannot be compared with existing composite drought indices due to missing data. Moreover, comparisons with other types of drought indices may make the assessment unfair and inaccurate, and ultimately lead to the masking of its shortcomings or strengths. Therefore, it is particularly essential to produce a high-resolution composite drought index dataset to compensate for the data deficiencies.

In this study, we constructed a 0.1° resolution global drought dataset (1980–2019) for MCDI and its constituent indices on a monthly scale. Specifically, we referred to the methodology of MCDI to first generate WSDI as well as SPAEI, SSI, and SRI at different time scales and then fused the indices to generate MCDI with accounting for the time lag and cumulative effects of drought. In this process, apart from the MCDI data, SPAEI, SSI, SRI, and WSDI data were generated as intermediates. Subsequently, we validated the credibility and usability of this dataset to provide effective data to support the development of drought management and adaptation strategies.

## Methods

### Data sources

The precipitation (P) and actual evapotranspiration (AET) data were used to produce SPAEI. Monthly P data for 1980–2019 were obtained from Multi-Source Weighted-Ensemble Precipitation (MSWEP). The P data have a spatial resolution of 0.1° and are available online (https://www.gloh2o.org/mswep/)^39–41^. Monthly AET data for the period 1980–2019 were sourced from the fourth generation of the Global Land Evaporation Amsterdam Model (GLEAM) with a spatial resolution of 0.1° (available online https://www.gleam.eu/)^42^.

The soil moisture (SM) data were used for calculating SSI. Monthly-scale SM for 1980–2019 was obtained from the ECMWF Reanalysis v5 (ERA5)-Land dataset (available online https://www.ecmwf.int/en/forecasts/dataset/ecmwf-reanalysis-v5-land) with a spatial resolution of 0.1°. The dataset contains SM data for four soil horizons (0–7 cm, 7–28 cm, 28–100 cm, and 100–289 cm), and SM in the 0–10 cm soil layer was weighted according to soil layer thickness^43,44^.

The runoff (RO) data were used to generate SRI. The monthly RO data were obtained from the ERA5-Land, Famine Early Warning Systems Network (FEWS NET) Land Data Assimilation System (FLDAS) (available online https://disc.gsfc.nasa.gov/datasets/FLDAS_NOAH01_C_GL_M_001/summary), and Ghiggi, *et al*.^45^ (available online https://figshare.com/articles/dataset/Grun_Global_Runoff_Reconstruction/9228176). Their spatial resolutions are 0.1°, 0.1°, and 0.5°, respectively. The bilinear interpolation^36,46,47^ was chosen in this study to resample the data to 0.1° to unify the spatial resolution. It is important to note that this interpolation process redistributes the original 0.5° data onto a finer grid but does not enhance the actual spatial detail of the source data. To further reduce the uncertainties from the source data, the final RO data for 1980–2019 at 0.1° resolution were generated by averaging the three sources.

The terrestrial water storage anomaly (TWSA) data were used for generating WSDI. Due to the requirement of temporal continuity and length of data, the reconstructed datasets of TWSA were selected for this study. The monthly TWSA data from 1980 to 2019 with a spatial resolution of 0.5° were obtained from Li, *et al*.^48^ (available online https://datadryad.org/dataset/doi:10.5061/dryad.z612jm6bt) and Humphrey and Gudmundsson^49^ (available online https://figshare.com/articles/dataset/GRACE-REC_A_reconstruction_of_climate-driven_water_storage_changes_over_the_last_century/7670849). To reduce uncertainties in the data sources, the final TWSA data were produced by averaging the three datasets before resampling to a 0.1° grid using bilinear interpolation. Similarly, bilinear interpolation merely distributes spatial information onto a finer-resolution grid without adding any new spatial information.

### MCDI calculation

As a comprehensive drought index, MCDI showed good ability to identify and characterize drought over China^30^. Therefore, referencing previous work, we developed a global high-resolution drought index dataset, which includes MCDI and its intermediates (SPAEI, SSI, SRI, WSDI). The generation of MCDI was mainly divided into three steps:

Firstly, calculating the drought indices (SPAEI, SSI, SRI, WSDI) involved in constructing MCDI. The standardized drought indices (SDI, including SPAEI, SSI, and SRI) share a common calculation principle. For a given time scale, the cumulative values of the underlying variable are fitted to a probability distribution and then converted to a standard normal distribution. Specifically, the SPAEI was derived by fitting the cumulative difference between P and AET to a Log-Logistic distribution^50,51^, whereas SSI and SRI were obtained by fitting cumulative SM, RO to a gamma distribution, respectively^9,18^. These indices were considered for time scales from 1 to 12 months, denoted as SDI1, SDI2,…, and SDI12. In contrast, WSDI was calculated directly by normalizing the water storage deficit (WSD) based on the mean and standard deviation of the WSD time series^52,53^. In which WSD was the deviation of the monthly TWSA from its long-term climatic mean^54^. Table 1 lists the drought classifications for SDI and WSDI.

**Table 1:** The drought categorization for drought indices.

Category	SPAEI, SSI, SRI, MCDI	scPDSI	WSDI
Normal	$\ge -0.5$	$\ge -1.0$	$\ge -0.5$
Slight Drought	$-1.0 \sim -0.5$	$-2.0 \sim -1.0$	$-1.0 \sim -0.5$
Moderate Drought	$-1.5 \sim -1.0$	$-3.0 \sim -2.0$	$-2.0 \sim -1.0$
Severe Drought	$-2.0 \sim -1.5$	$-4.0 \sim -3.0$	$-3.0 \sim -2.0$
Extreme Drought	$\le -2.0$	$\le -4.0$	$-4.0 \sim -3.0$

Secondly, calculating the lag time between different types of droughts at the pixel scale and regenerating drought indices data based on the lag time. Specifically, for meteorological and hydrological droughts, we calculated the Pearson correlation coefficient between the hydrological drought index (WSDI) and the meteorological drought index (SPAEI) across cumulative timescales from 1 to 12 months (SPAEI-n, n = 1, 2,…, 12). The lag time for a given pixel was definitively identified as the specific timescale n (months) at which this correlation coefficient reached its maximum value. Then we created the new dataset (SPAEI_new_) by mapping each pixel to the drought index value at its specific lag timescale. For example, in the resulting SPAEI_new_ dataset, a pixel with a 3-month lag time was assigned the SPAEI3 value. This method was subsequently repeated to construct the SSI_new_ and SRI_new_ datasets.

Finally, the MCDI was constructed based on the regenerated drought index dataset and the Gringorten empirical joint probability distribution^55^. For a total of N = 480 monthly observations, the joint empirical probability ${{p}}_{i}$ for the i-th observation was calculated as ${(m}_{i}-0.44)/(N+0.12)$. Here, ${m}_{i}$ counted how often all four indices were simultaneously at or below their i-th values. Then, MCDI was computed by transforming ${{p}}_{i}$ using the inverse standard normal distribution.

**Fig. 1:**
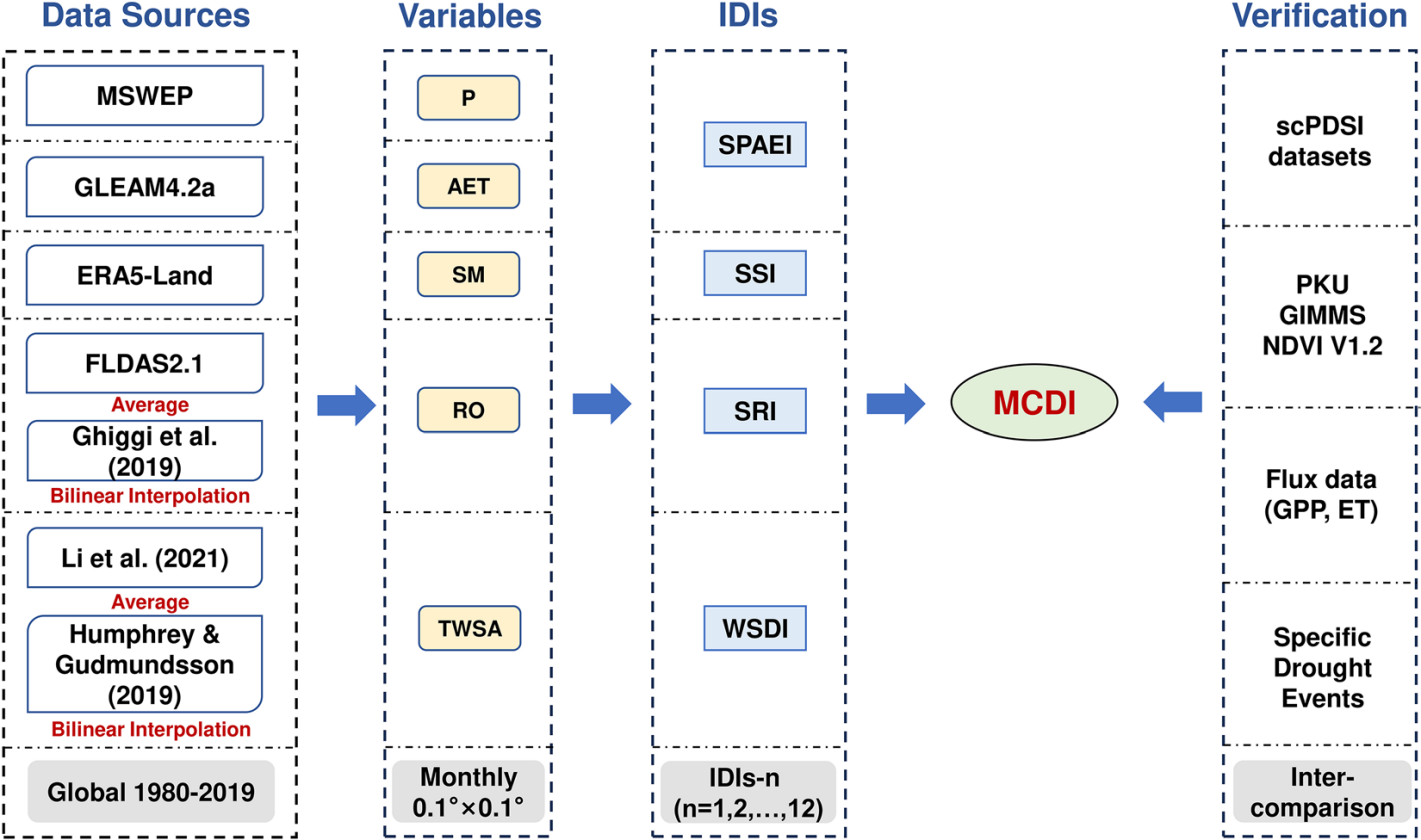
The flowchart for the global drought index dataset generation and validation. P, AET, SM, RO, and TWSA represent precipitation, actual evapotranspiration, soil moisture, runoff, and terrestrial water storage anomaly, respectively. IDIs represent intermediate drought indices.

A more detailed calculation procedure can be found in Yuan, *et al*.^30^. The drought classification of MCDI is shown in Table 1. The datasets, variables involved in developing MCDI, and the process of generating and validating MCDI are shown in Fig. 1.

### Evaluation criteria

The self-calibrating Palmer Drought Severity Index (scPDSI)^34^ is one of the most recognized and widely used drought indices at present. Compared with other drought indices, scPDSI involves multiple variables in the water cycle process and is relatively more comprehensive. Therefore, this study used the existing scPDSI dataset to assess the performance of MCDI and its constituent indices (SPAEI, SSI, SRI, WSDI). The monthly scPDSI data from 1980 to 2019 with a spatial resolution of 0.5° were available online https://crudata.uea.ac.uk/cru/data/drought/^32,33^. To harmonize the spatial resolution of the data, scPDSI data were resampled to 0.1° using bilinear interpolation, which merely redistributes the data onto a finer grid without adding spatial detail.

The Normalized Difference Vegetation Index (NDVI) was used to evaluate the performance of MCDI on the global scale. Furthermore, it helped explore the consistency of MCDI and ecosystem responses to droughts. The NDVI data were obtained from the PKU GIMMS Normalized Difference Vegetation Index product (PKU GIMMS NDVI, version 1.2) (available online https://zenodo.org/records/8253971)^56^. Based on a machine learning model, PKU GIMMS NDVI integrated Landsat Surface Reflectance data, the GIMMS NDVI3g (V1.0) product, and the MODIS Vegetation Index product. Accuracy validation results showed that PUK GIMMS NDVI effectively removed the evident orbital drift and sensor degradation effects in the tropics. PKU GIMMS NDVI has a spatial resolution of 1/12° and a temporal resolution of half a month. To unify the resolution, we averaged the two images for each month to obtain the monthly mean and resampled the data to 0.1° using bilinear interpolation. It is acknowledged that this process results in a spatial degradation and a smoothing of the original high-resolution information.

Flux data obtained from FLUXNET (https://fluxnet.org/data)^57^ were used to further assess the validity of this drought dataset. We first filtered out 30 flux sites by several conditions: (1) data were available for at least 10 years; (2) Gross primary productivity (GPP) and ET data were available; (3) all drought indices had valid values at this site. Then we standardized GPP and ET by subtracting their multi-year contemporaneous means and dividing by their standard deviations. Details of the flux sites are shown in Table 2.

**Table 2:** The description of 30 flux sites used in this study.

Site	IGBP	Lon (°)	Lat (°)	Data availability
AT-Neu	GRA	11.32	47.12	2002–2012
AU-How	WSA	131.15	−12.49	2001–2014
AU-Tum	EBF	148.15	−35.66	2001–2014
BE-Lon	CRO	4.75	50.55	2004–2014
BE-Vie	MF	6.0	50.31	1996–2014
CH-Dav	ENF	9.86	46.82	1997–2014
DE-Geb	CRO	10.91	51.1	2001–2014
DE-Gri	GRA	13.51	50.95	2004–2014
DE-Hai	DBF	10.45	51.08	2000–2012
DE-Tha	ENF	13.57	50.96	1996–2014
FI-Sod	ENF	26.64	67.36	2001–2014
FR-LBr	ENF	−0.77	44.72	1996–2008
FR-Pue	EBF	3.56	43.74	2000–2014
IT-BCi	CRO	14.96	40.52	2004–2014
IT-Lav	ENF	11.28	45.96	2003–2014
IT-MBo	GRA	11.05	46.01	2003–2013
NL-Loo	ENF	5.74	52.17	1996–2014
RU-Fyo	ENF	32.92	56.46	1998–2014
US-Blo	ENF	−120.63	38.9	1997–2007
US-GLE	ENF	−106.24	41.37	2004–2014
US-Me2	ENF	−121.56	44.45	2002–2014
US-MMS	DBF	−86.41	39.32	1999–2014
US-Ne1	CRO	−96.48	41.17	2001–2013
US-Ne2	CRO	−96.47	41.16	2001–2013
US-Ne3	CRO	−96.44	41.18	2001–2013
US-NR1	ENF	−105.55	40.03	1998–2014
US-SRM	WSA	−110.87	31.82	2004–2014
US-Ton	WSA	−120.97	38.43	2001–2014
US-Var	GRA	−120.95	38.41	2000–2014
US-Wkg	GRA	−109.94	31.74	2004–2014

IGBP = International Geosphere Biosphere Programme, CRO = Croplands, EBF = Evergreen broadleaf forests, ENF = Evergreen needleleaf forests, GRA = Grasslands, MF = Mixed forests, WSA = Woody Savannas.

Apart from the above four evaluation indicators, we also selected three typical drought events to assess the ability of MCDI to monitor drought. These events caused relatively large losses and attracted widespread attention, occurring in Australia, the Contiguous United States, and South Africa, respectively. For Australia, 2019 was the driest year on record^58^. The widespread drought in Australia was primarily driven by excessive temperatures and persistent precipitation deficits that in 2017^59^. In 2019, temperatures were 1.52 °C above the multi-year average, and a precipitation deficit from April to November prevented soil moisture and runoff generation^60,61^. This extreme drought had led to massive canopy dieback and record-breaking wildfire events in southeastern Australia^62^. For the Contiguous United States, the drought that happened in 2012 was one of the most severe drought events since 1950^63^. It began during the growing season and affected 80% of the agricultural land in the Contiguous United States, causing not only significant economic losses but also a major impact on food security and food prices^64^. For South Africa, between December 2015 and February 2016, a severe drought event triggered by El Niño occurred^65^. Statistically, this drought event reached the highest extreme temperatures in the past 50 years^66^. In addition, the drought also had a significant impact on food production. The model simulations showed that this drought event led to the largest reduction in food production over the past 30 years^67^.

### Statistical methods

In geographical and hydrological research, Pearson correlation coefficients (PCC) are often used to analyze the degree of correlation between variables and to evaluate the accuracy of a variable against a reference variable^68^. In this study, the PCC between scPDSI/NDVI/GPP/ET and MCDI, as well as intermediate drought indices (SPAEI, SSI, SRI, WSDI), was calculated separately to assess their credibility and usability. The strength of the correlation was interpreted based on the absolute value of the PCC (|PCC|). Within the context of drought indices, correlations commonly fall in the 0.3~0.5 range, and values above 0.5 are generally regarded as indicating a relatively strong association^69–73^. Accordingly, we adopted the explicit criteria from Wang, *et al*.^74^. The correlations were classified as follows: |PCC| ≥ 0.8 for extremely strong, 0.8 > |PCC| ≥ 0.5 for strong, 0.5 > |PCC| ≥ 0.3 for medium, and |PCC| < 0.3 for weak correlation. Consequently, |PCC| = 0.3 and |PCC| = 0.5 were employed as the two key thresholds in this study.

## Data Records

The dataset for the MCDI and indices used to construct the MCDI (SPAEI, SSI, SRI, WSDI) is openly available on the Zenodo repository under the Creative Commons Attribution 4.0 International (CC BY 4.0) license (10.5281/zenodo.15143908^75^). The high-resolution dataset has a spatial resolution of 0.1° × 0.1°, a spatial range of 180°W to 180°E and 90°S to 90°N, a period of 1980–2019, and a temporal resolution of monthly scale. The dataset is presented in geographic latitude/longitude projection (EPSG: 4326) and stored using the NetCDF format. A total of 38 data files are included in the dataset, with drought index names in the file names. For SPAEI, SSI, and SRI with multi-timescale characteristics, the drought index names are followed by the timescale, e.g., SPAEI3/SSI3/SRI3.

## Technical Validation

### Agreement between MCDI (SPAEI, SSI, SRI, WSDI) and scPDSI

**Fig. 2:**
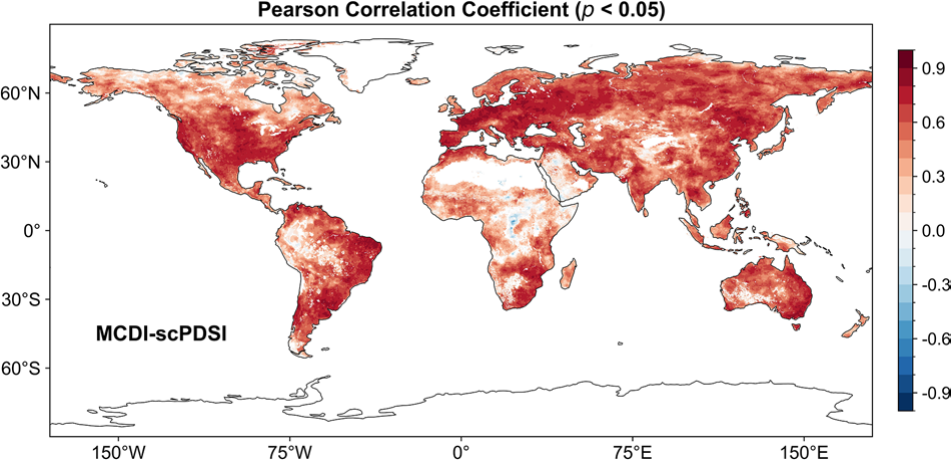
The spatial distribution of the Pearson correlation coefficient (*p* < 0.05) for the MCDI and scPDSI over the period 1980–2019.

Figure 2 presents the spatial distribution of the Pearson correlation coefficients (*p* < 0.05) for the MCDI and scPDSI over the period 1980–2019. MCDI and scPDSI exhibited similar spatial distribution characteristics with good correlation. To quantify their agreement, we used |PCC| = 0.3 and |PCC| = 0.5 as thresholds, corresponding to medium and strong correlation levels, respectively. Although these criteria were applied symmetrically to both positive and negative correlations, the spatial proportion of pixels with significant negative correlations (PCC ≤ −0.3) was negligible (consistently below 0.5% in all comparisons). Therefore, we focus on the dominant positive correlation patterns here. Globally, the proportion of pixels with PCC greater than 0.5 between MCDI and scPDSI was 59.84% (*p* < 0.05), while the proportion of pixels with PCC greater than 0.3 was as high as 86.69% (*p* < 0.05). The mean values of the PCC between MCDI and scPDSI in North America, South America, Europe, Asia, Africa, and Oceania were 0.46, 0.54, 0.61, 0.51, 0.40, and 0.56 (*p* < 0.05), respectively. Spatially, the areas with relatively lower correlation between MCDI and scPDSI were mainly concentrated in tropical regions and high-latitude regions, such as tropical Africa and northern North America, which has also been found in other studies^65,76^. This phenomenon may be because these two regions are susceptible to factors such as large-scale circulation, resulting in the inability of the aridity index to correctly capture the characteristics of wet and dry dynamics. In addition, the susceptibility of sparsely vegetated areas in Africa to surface radiation may lead to poorer quality source data from satellite observations, transferring errors to the drought index, which ultimately results in poorer correlations among the drought indices^65,77^.

**Fig. 3:**
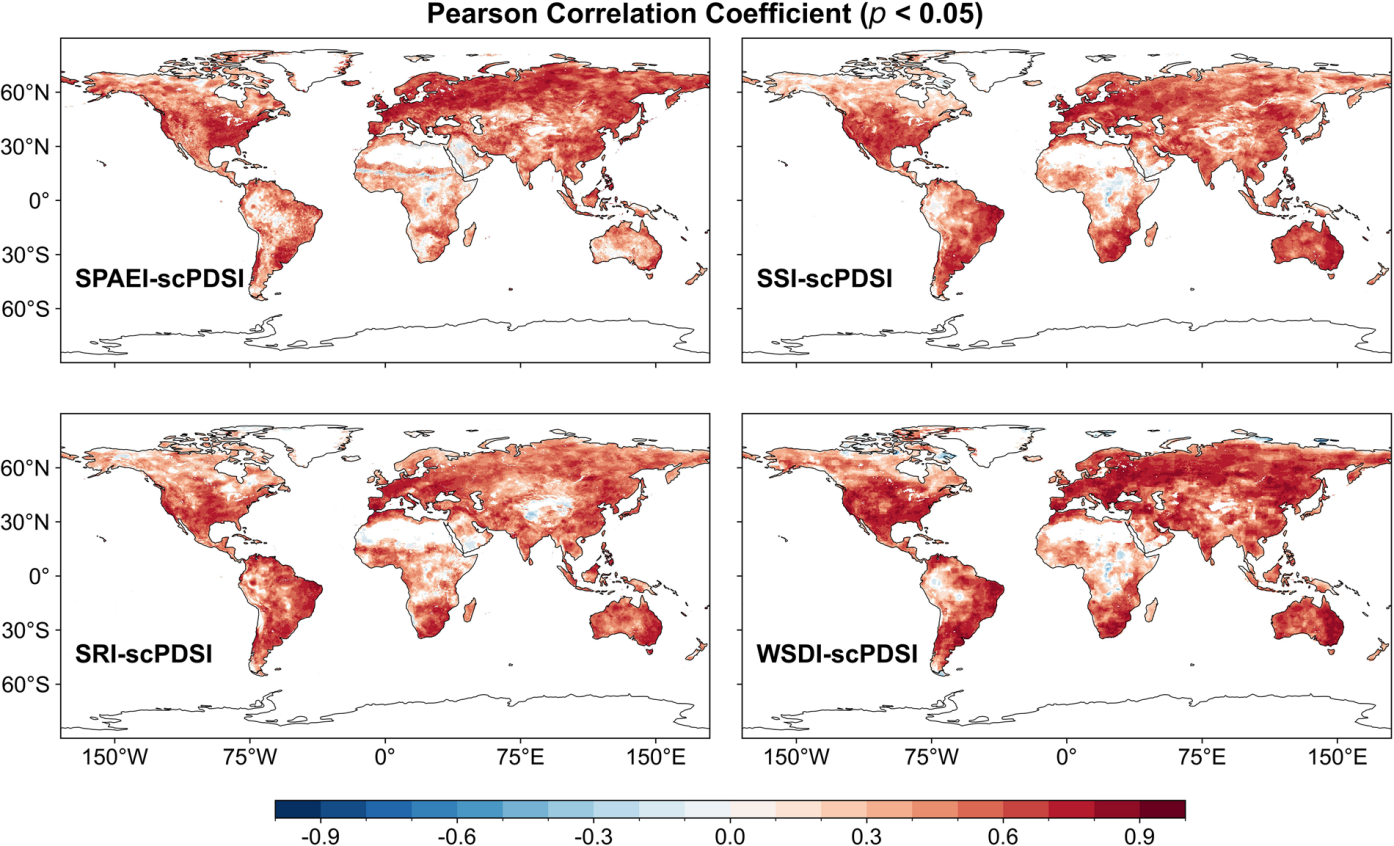
The spatial distribution of the maximum Pearson correlation coefficient (*p* < 0.05) between scPDSI and SPAEI, SSI, and SRI on different time scales (1–12 months) and the correlation between scPDSI and WSDI over the period 1980–2019.

Figure 3 displays the maximum correlation between scPDSI and SPAEI/SSI/SRI on different time scales and the correlation between scPDSI and WSDI. The percentages of pixels with PCC greater than 0.5 for SPAEI, SSI, SRI, and WSDI were 50.38%, 50.34%, 47.69%, and 59.34% (*p* < 0.05), respectively. The proportion of pixels with PCC greater than 0.3 between SPDSI and SPAEI, SSI, SRI as well as WSDI all exceeded 80.0% but was lower than 84.0% (*p* < 0.05). Even though the spatial distribution of correlations between different drought indices (SPAEI, SSI, SRI, and WSDI) and scPDSI showed similar characteristics, the correlation values between them varied in different regions. This result may be due to fundamental differences in construction principles or dependent variables between drought indices^78^.

Although drought indices in this dataset all showed good correlations with the scPDSI, the MCDI presented some superiority compared to the others. The percentage of pixels with correlations greater than 0.5 for MCDI and scPDSI was 9.46%, 9.51%, 12.16%, and 0.51% (*p* < 0.05) higher than SPAEI, SSI, SRI, and WSDI, respectively. In addition, the mean values of the correlations of MCDI and scPDSI in different continents were always higher than other drought indices or lower than others by a small difference. The possible reason for this phenomenon is that MCDI has the advantage of being a comprehensive drought index by considering multiple components of the water balance theory^30^. While other drought indices are constructed based on different components with their focus, this fundamental difference may lead to their inherent limitations in characterizing drought^79^.

### Agreement between MCDI (SPAEI, SSI, SRI, WSDI) and NDVI

**Fig. 4:**
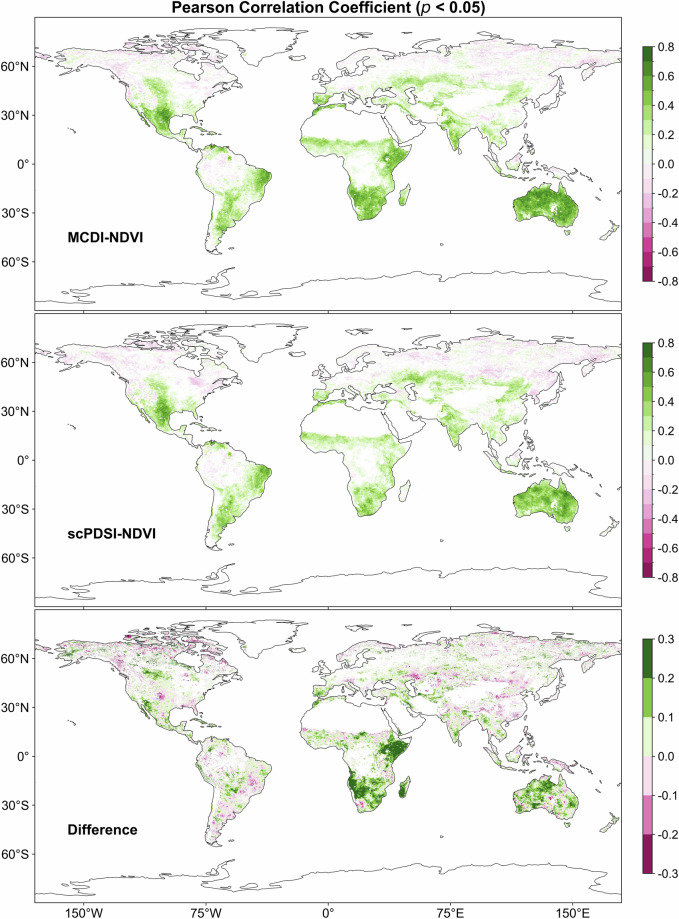
The spatial distribution of the Pearson correlation coefficient (*p* < 0.05) between MCDI/scPDSI and NDVI as well as their difference over the period 1982–2019.

Figure 4 presents the PCC between MCDI/scPDSI and NDVI as well as the difference between them. Overall, both MCDI and scPDSI showed relatively high positive correlations with NDVI in southern Africa, inland Australia, eastern and southern South America, southern North America, and southern Asia. In some tropical rainforests, perennial high-humidity areas (e.g., Central African rainforests, parts of the Amazon Basin), and high-latitude areas (e.g., Northern Europe and Asia), the correlation between MCDI/scPDSI and NDVI was relatively low or even sporadically negative. Apart from the quality of the source data, this may be because vegetation growth in these areas is also affected by temperature, radiation, or other factors, not just water constraints^3^. In other words, it is the complexity of vegetation physiological processes and other climatic and environmental drivers that cause non-synergistic variations in vegetation and drought indices^65,76^. Compared to scPDSI, MCDI presented higher PCC in some regions. The proportion of pixels with PCC greater than 0.3 between MCDI and NDVI was 31.89% (*p* < 0.05), while scPDSI was only 23.99% (*p* < 0.05). The differences in the correlation between the two and NDVI were mainly found in regions such as southern and eastern Africa and Australia. It indicated that the explanatory power of MCDI for NDVI was generally higher than that of scPDSI in these regions and that MCDI was better able to capture the response of vegetation to wet-dry changes, also suggesting its greater applicability and stability in capturing multifactorial moisture dynamics. As for the reason, it may be that NDVI has a lag in its response to the environment, and the MCDI was constructed in such a way as to account for the lag and cumulative effects of environmental and climatic conditions.

**Fig. 5:**
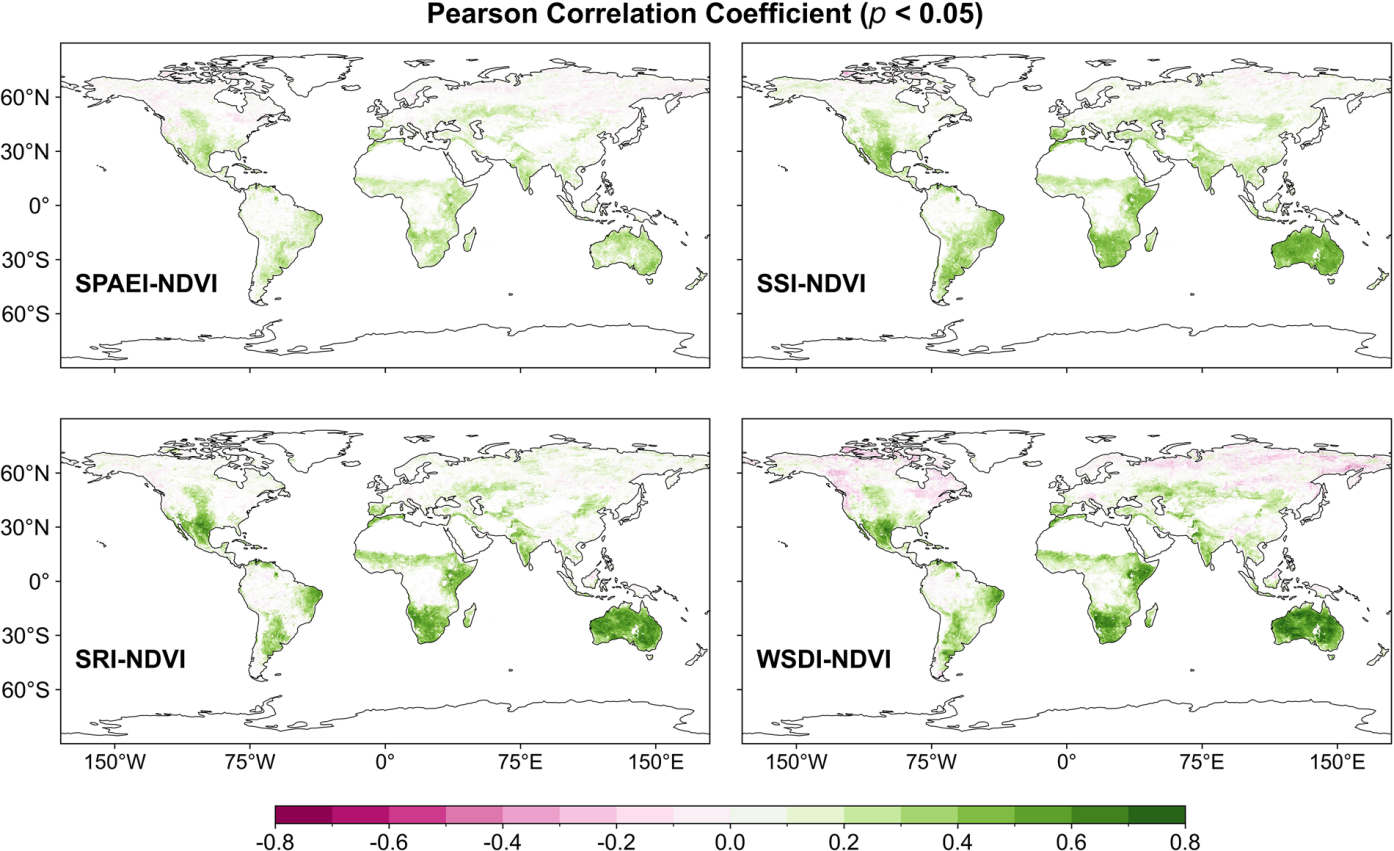
The spatial distribution of the Pearson correlation coefficient (*p* < 0.05) between NDVI and WSDI and the maximum Pearson correlation coefficient (*p* < 0.05) with SPAEI, SSI, and SRI at different time scales (SPAEI-n/SSI-n/SRI-n (n = 1, 2,…, 12)) over the period 1982–2019.

Figure 5 illustrates the correlation between NDVI and WSDI and the maximum correlation with SPAEI, SSI, and SRI at different time scales (SPAEI-n/SSI-n/SRI-n (n = 1, 2,…, 12)). The percentages of pixels with PCC greater than 0.3 for SPAEI, SSI, SRI, and WSDI were 22.38%, 45.59%, 37.61%, and 30.01% (*p* < 0.05), respectively. It can be found that the spatial distribution of the correlations presented by the four drought indices with NDVI was like that of MCDI and scPDSI. They showed significant positive correlations with NDVI in regions such as inland Australia and Central Asia, while they had low or even negative correlations at high latitudes and in regions with overabundant thermal and hydrological conditions. That is, like MCDI and scPDSI, they were also effective in capturing ecosystem responses to changes in the external environment, though there were some differences among local areas.

### Agreement between MCDI (SPAEI, SSI, SRI, WSDI) and measured flux data

**Fig. 6:**
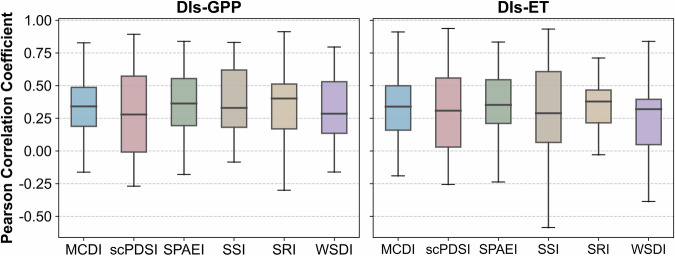
The Pearson correlation coefficients between the drought indices (MCDI, SPAEI, SSI, SRI, WSDI, scPDSI) and measured flux data (GPP, ET) at 30 flux sites during droughts.

GPP and ET are commonly used to indicate ecosystem status^80,81^. Figure 6 presents the Pearson correlation coefficients between the drought indices (MCDI, scPDSI, SPAEI, SSI, SRI, WSDI) and measured flux data (GPP, ET) at 30 flux sites during droughts. The mean PCC values of drought indices (MCDI, scPDSI, SPAEI, SSI, SRI, WSDI) and GPP at 30 flux sites were 0.33, 0.27, 0.31, 0.36, 0.34, and 0.30, respectively. Among them, MCDI exhibited a more concentrated data distribution profile, which may be attributed to the fact that MCDI integrates multiple meteorological and hydrological factors^30^. In contrast, although scPDSI also considers multiple variables of the water cycle, it performed poorly with high variance. This may be due to the fact that scPDSI does not account for the long-term cumulative effect of drought and the lagged response of ecosystems, which ultimately leads to non-synergistic changes in scPDSI and GPP^26,47,82^, whereas MCDI makes up for this deficiency. Moisture is a key limiting factor for plant photosynthesis, and soil moisture and runoff affect GPP through direct water supply and resource reallocation, respectively^44,77,83^. Thus, the phenomenon of SSI and SRI showing a favorable correlation with GPP was also explained. The average PCC values of drought indices (MCDI, scPDSI, SPAEI, SSI, SRI, WSDI) and ET at 30 flux sites were 0.34, 0.31, 0.35, 0.33, 0.30, and 0.26, respectively. Due to the close linkage between GPP and ET, the correlation between drought indices and ET showed similar characteristics^84^. The main reason for the superior correlation between SPAEI and ET over other indices was that the SPAEI considered actual evapotranspiration, and the MCDI, which incorporated the SPAEI, similarly reflected this advantage. Overall, validation with measured flux data showed that the changes in MCDI (SPAEI, SSI, SRI, WSDI) were consistent with the ecosystem response to droughts, and MCDI were more robust compared to other indices.

### Performance of drought indices (MCDI, SPAEI, SSI, SRI, WSDI) during specific drought events

**Fig. 7:**
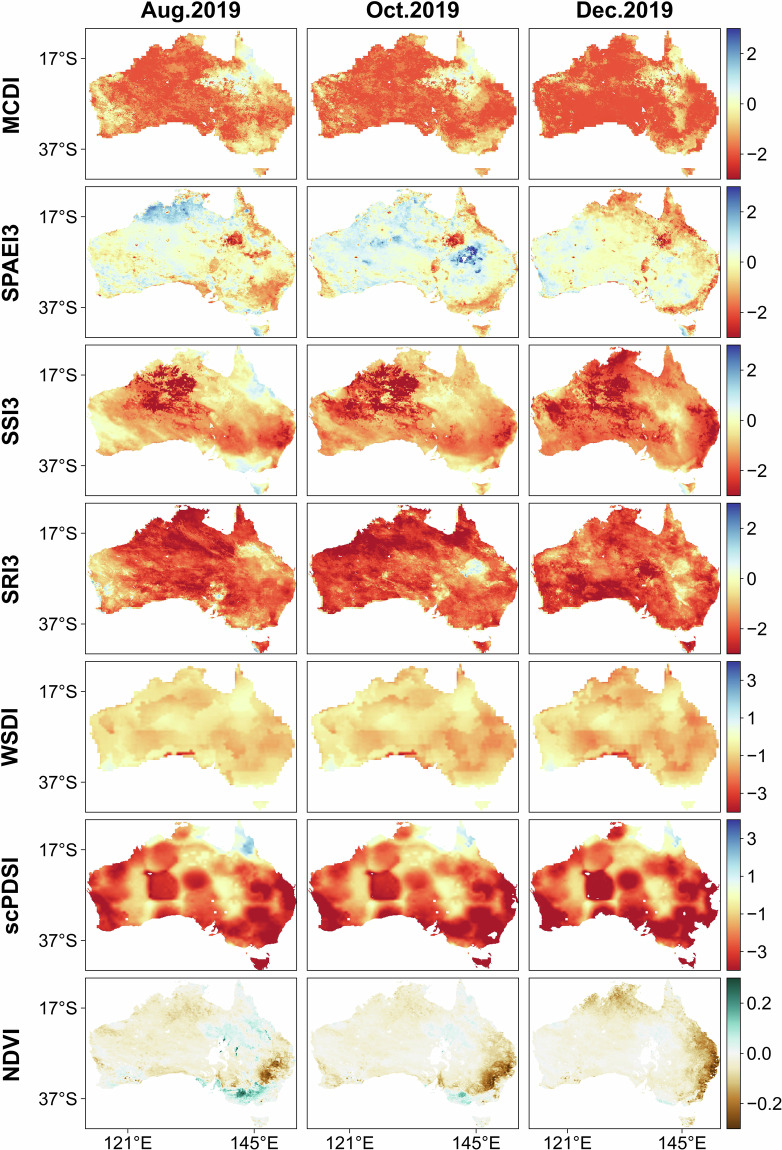
The spatial and temporal dynamics of MCDI, the constituent indices of MCDI (SPAEI3, SSI3, SRI3, WSDI), scPDSI, and NDVI for August, October, and December 2019 in Australia.

Figure 7 presents the spatial and temporal dynamics of MCDI, the constituent indices of MCDI (SPAEI3, SSI3, SRI3, WSDI), scPDSI, and NDVI in Australia for August, October, and December 2019. Australia was in a persistent drought from August to December, with a peak of drought severity (drought extent and drought index values) in December. The NDVI showed a decline in vegetation productivity in December in almost all regions, particularly in eastern Australia. Correspondingly, the scPDSI presented drought conditions across almost all of Australia, particularly in the southeastern and southern regions. As we know, the drought in 2019 was caused by the accumulation of pre-drought environmental conditions, and at this point, the type of drought might have switched to one dominated by agricultural and hydrological drought^58,59^. This might also be the reason why SPAEI3 showed lower drought extent and severity compared to other drought indices. Soil moisture and runoff were not recharged for a long period, so it was reasonable that SSI3 and SRI3 both exhibited severe drought status. WSDI characterizes changes in terrestrial water storage, accounting for a combination of variables such as surface water and groundwater^85^. Although WSDI values were relatively lower and less variable during drought, it was equally effective in indicating drought conditions. MCDI integrated SPAEI, SSI, SRI, and WSDI, showing their combined characteristics. Thus, the differences presented by different drought indices in characterizing the same drought event and the extremes exhibited by a single drought index were effectively avoided.

**Fig. 8:**
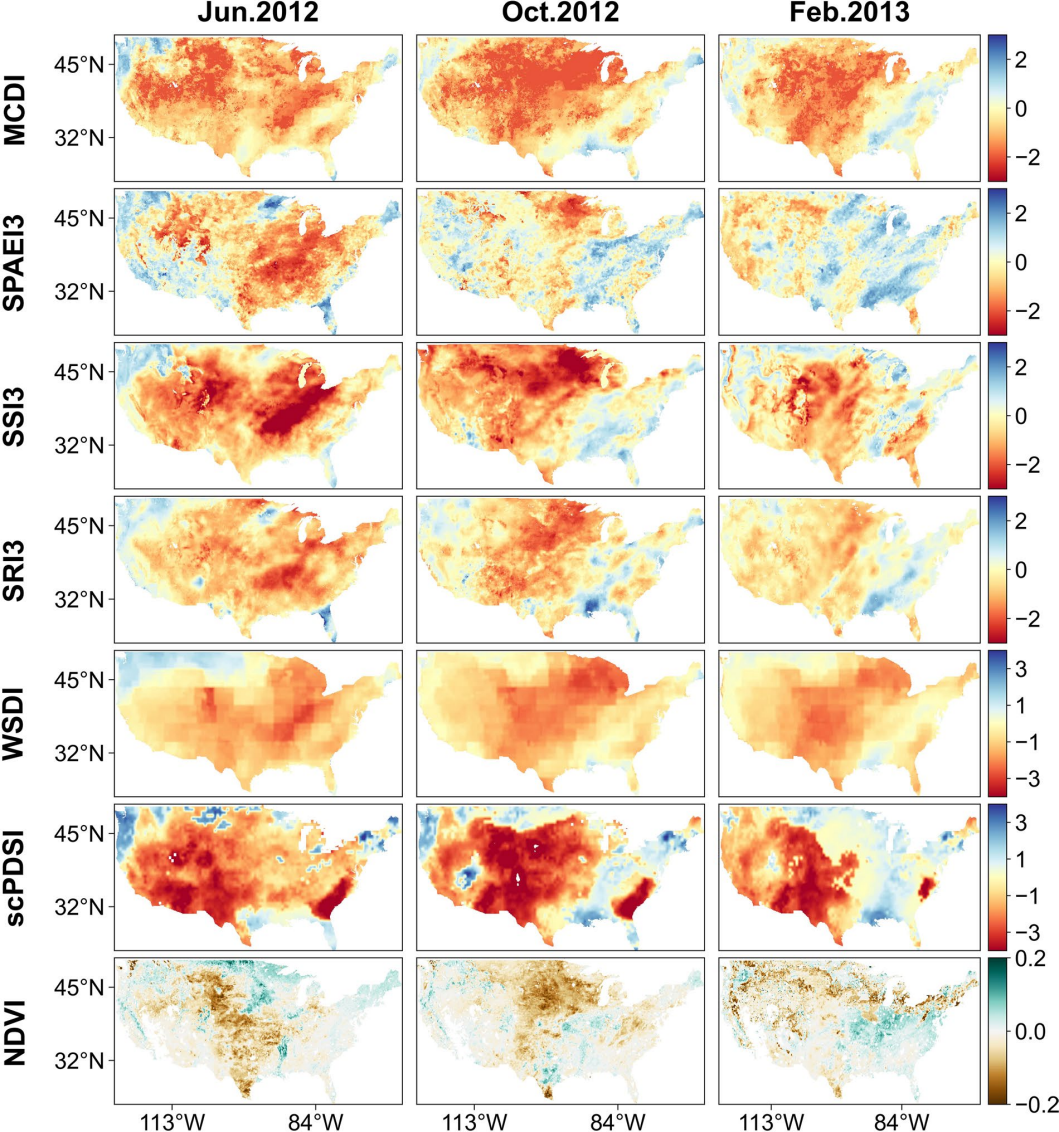
The spatial and temporal dynamics of MCDI, the constituent indices of MCDI (SPAEI3, SSI3, SRI3, WSDI), scPDSI, and NDVI for June and October 2012, and February 2013 in the Contiguous United States.

Figure 8 displays the spatial and temporal dynamics of MCDI, the constituent indices of MCDI (SPAEI3, SSI3, SRI3, WSDI), scPDSI, and NDVI during the drought event that happened in the Contiguous United States. During the period from June 2012 to February 2013, the drought severity continued to decrease, and the extent of drought gradually narrowed from the global state to the Midwest. By October 2012, a significant decline in NDVI was observed in the central Contiguous United States, which is dominated by agricultural cultivation, suggesting that crops in this region were severely affected by the drought. In the subsequent months up to February 2013, productivity in the northern and western regions was also shown to be impacted. However, scPDSI seemed not to be successful in indicating the drought status of these regions across this period. In contrast, the drought status indicated by the MCDI and the vegetation growth status indicated by the NDVI were in better agreement. In addition, this drought event led to increased water use, and ultimately, groundwater declined due to the lack of precipitation and prolonged soil moisture deficits, which also might be the reason why the WSDI still indicated drought in the eastern part of the region in February 2013^86,87^.

**Fig. 9:**
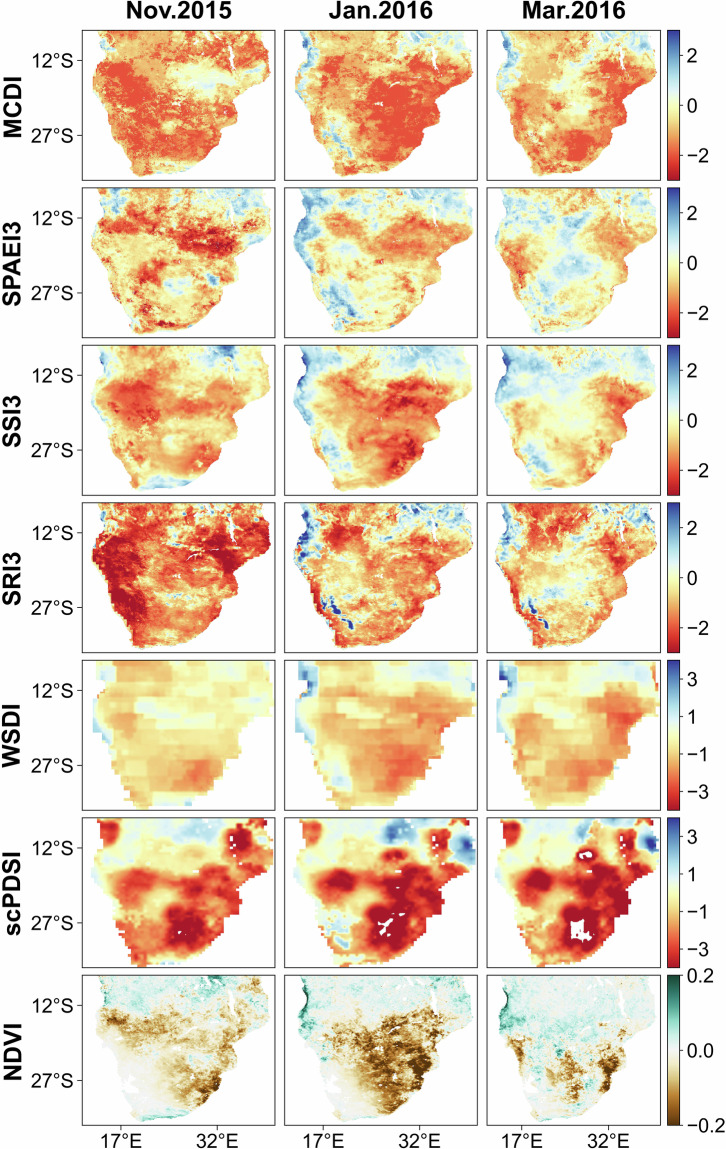
The spatial and temporal dynamics of MCDI, the constituent indices of MCDI (SPAEI3, SSI3, SRI3, WSDI), scPDSI, and NDVI for November 2015, January 2016, and March 2016 in South Africa.

Figure 9 shows the spatial and temporal dynamics of MCDI, the constituent indices of MCDI (SPAEI3, SSI3, SRI3, WSDI), scPDSI, and NDVI for November 2015, January 2016, and March 2016 in South Africa. The decreasing NDVI over a wide area from November 2015 to January 2016 signified that the drought had a large impact on vegetation productivity within South Africa. By March 2016, the area with NDVI affected gradually narrowed down to parts of the eastern and central regions. However, at this time, the drought indices still indicated widespread drought conditions, especially MCDI and scPDSI. The reason for this phenomenon might be that the improvement of NDVI may result from occasional light rainfall or drought adaptation of vegetation, while the drought indices reflect a combination of available water and climatic conditions^76^. Due to the El Niño phenomenon, where high temperatures and moisture deficits co-existed, the drought indices were influenced by historical cumulative water deficits and thus still indicated drought conditions. In addition, despite the differences in spatial and temporal performance between the different indices, it must be recognized that all drought indices captured the characteristics of drought events well.

## Data Availability

The dataset is available at 10.5281/zenodo.15143908.

## Code availability

The code used to synthesize results and replicate all the figures in this manuscript can be accessed from the link https://zenodo.org/records/16343702. The scripts were written in Python version 3.9.7 (https://www.python.org/).
